# Effects of Vacuolar H^+^-ATPase Inhibition on Activation of Cathepsin B and Cathepsin L Secreted from MDA-MB231 Breast Cancer Cells

**DOI:** 10.1007/s12307-017-0196-7

**Published:** 2017-08-02

**Authors:** Andrew Uhlman, Kelly Folkers, Jared Liston, Harshida Pancholi, Ayana Hinton

**Affiliations:** Biology Department, Denison University, 100 W. College St, Granville, OH 43023 USA

**Keywords:** Vacuolar H^+^-ATPase, Cysteine cathepsins, Breast cancer cells, Tumor microenvironment, Protease activation

## Abstract

Studies indicate secreted cathepsins are involved in metastasis. V-ATPases, which are necessary for activating intracellular cathepsins, also play a role in metastasis and are targeted to the plasma membrane of metastatic breast cancer cells. We are interested in a connection between cell surface V-ATPases, activation of secreted cathepsins and the metastatic phenotype of MDA-MB231 cells. We investigated whether V-ATPase inhibition would reduce the activity of secreted cathepsin B and cathepsin L. Using cell lysates and conditioned media, we measured cathepsin B and L activity within and outside of the cells. We found different forms of cathepsin B and L were secreted representing the pre-pro, pro and active forms of the proteases. Cathepsin B activity was higher than cathepsin L in conditioned media and in cell lysates. V-ATPase inhibition by concanamycin A decreased cathepsin B activity in conditioned media and significantly decreased cathepsin B activity in cell lysates. Cathepsin L activity showed a slight decrease in cell lysates. Changes in the activity of secreted and intracellular cathepsins following V-ATPase inhibition were supported by changes in the amounts of pro and active forms of cathepsin B in conditioned media and cathepsins B and L in cell lysates. Overall, our data shows that inactive forms of cathepsins B and L are secreted from the MB231 cells and V-ATPase activity is important for the activation of secreted cathepsin B. This indicates a connection between cell surface V-ATPases in metastatic breast cancer cells and the function of secreted cathepsin B.

## Introduction

The leading cause of death from cancer is metastasis [1]. Inhibition of metastasis represents an important means of improving the prognosis for patients with cancer and identification of novel drug targets is critical to achieving this goal. Key steps to the successful spread of a tumor from an initial site of growth to other tissues is the loss of adhesion to surrounding cells and underlying stroma and the degradation of extracellular matrix to facilitate invasion and migration. Many proteins have been identified as playing a role in these steps including Vacuolar H^+^-ATPases (V-ATPases) and various members of the family of cysteine cathepsins. Cysteine cathepsins have been intensively investigated as potential targets for drugs aimed at slowing metastasis [2]. V-ATPases have also been proposed as possible drug targets to treat cancer metastasis as well as other hallmark features of cancer cells [3, 4]. Interestingly these proteins are fundamentally linked; intracellularly the activity of cysteine cathepsins depends on the activity of V-ATPases [4].

V-ATPases have been shown to function in metastasis of many cancer types including breast cancer. However little is known about the mechanisms of invasion mediated by V-ATPases. The V-ATPases are a family of ATP-dependent proton pumps whose primary role is in acidification of intracellular compartments, where they function in such processes as macromolecule degradation, receptor-mediated endocytosis and vesicular trafficking [5]. V-ATPases are also critical for bone resorption by osteoclasts, acid-base balance in the kidney, sperm maturation, insulin secretion and neurotransmitter uptake [5]. V-ATPases are upregulated in tissue samples from highly invasive pancreatic carcinomas [6] and treatment of a human cancer cell line with anti-sense oligonucleotides to the V-ATPase c subunit decreased invasion in vitro [7]. Inhibition of V-ATPase expression in hepatocellular carcinoma cells using siRNAs reduces invasiveness of these cells in vitro and metastasis in vivo [8]. V-ATPases are found intracellularly in nearly all cells, but only a few specialized cells (such as osteoclasts) have V-ATPases in their plasma membrane. However, V-ATPases have been identified in the plasma membrane of highly metastatic MDA-MB231 (MB231) human breast cancer cells but not in poorly metastatic MCF7 breast cancer cells [9]. Treatment of MB231 cells with specific inhibitors of the V-ATPase has been shown to block invasiveness of MB231 cells in vitro [9, 10]*.* The mechanism by which plasma membrane V-ATPases might be contributing to a metastatic phenotype has not been worked out, but it has been proposed that cell surface V-ATPases may work by activating lysosomal proteases that are secreted by invasive cancer cells [10].

Cysteine cathepsins are a family of proteases that prototypically reside within the lysosome where they function in the degradation and recycling of proteins. Some of the members of the cysteine cathepsin family also have more specific roles within certain cell types [11]. The family of cysteine cathepsins consists of 11 proteins in humans [12] and several different cysteine cathepsins have been found in cancer cells either associated with the cell membrane or secreted from the cells [13]. Cathepsin B (Cat B) and cathepsin L (Cat L) have been repeatedly studied for their role in tumorigenesis and metastasis. Increased expression of cathepsin B has been identified as a marker for poor prognosis for cancer survival [14, 15]. Inhibiting cathepsin B has been shown to limit metastasis to bone and lungs in breast cancer cells [16, 17]. Cathepsin L secretion is associated with late stage and malignant tumors [18, 19]. Both cathepsin B and cathepsin L are known to activate extracellular proteases that degrade extracellular matrix proteins [12, 20]. There is also in vitro evidence that cathepsin B and cathepsin L can directly degrade components of the extracellular matrix [19, 21]. In addition, cathepsin L has been shown to degrade E-cadherin an important cell adhesion protein [19].

We wanted to investigate whether either of these cathepsins might be working with the V-ATPase to facilitate invasion by human breast cancer cells. We hypothesized that by inhibiting V-ATPases the activity of one or both of these cathepsins in the extracellular space (conditioned media) would decrease. This should be due to two factors: 1) inhibiting the V-ATPase will effect vesicular trafficking thus affecting secretion and more interestingly 2) inhibiting cell surface V-ATPases will lead to decreased processing of the secreted pro form of the cathepsins to the active form thus leading to less cathepsin activity in the extracellular space.

## Materials and Methods

### Cell Culture

MDA-MB231 (MB231) breast cancer cells (ATCC) were grown at 37 °C at 7% CO_2_ in Dulbecco’s Modified Eagle’s media (DMEM) with phenol, penicillin-streptomycin, non-essential amino acids and 10% newborn calf serum (NBCS). This will be designated as “DMEM complete” media hereafter.

### Activity Assay

#### Treatment with Concanamycin A

Cells plated at 2 X 10^6^ cells/ml in T-75 flasks were grown for DMEM complete for 48 h at which point the medium was replaced with DMEM without phenol and concanamycin A (ConA) was added to the flask to 100 nM. An equivalent volume of dimethyl sulfoxide (DMSO) was added to control cells. After 24 h the conditioned medium was recovered from the flask, concentrated ~40 times in 10 K cut-off Amicon® Ultra centrifugal filters from Millipore and frozen for later assays. Treated cells were harvested via trypsinization and then cells were spun down, resuspended in 500ul PBS + 1% Triton X-100, lysed, centrifuged and the supernatant was frozen for later assays. Protein concentration for all samples was determined using Pierce BCA protein assay kit.

#### Activity Assay

The BioVision Cathepsin Activity Fluorometric Assay kits for Cathepsin B and Cathepsin L were used to measure the cathepsin activity of each sample. The cathepsin L kit uses the fluorogenic substrate Z-Phe-Arg-AFC (amino-4-trifluoromethyl coumarin) and the cathepsin B kit uses Z-Arg-Arg-AFC. 10 µl of conditioned media or cell lysate sample was assayed following the manufacturer’s recommended protocol. Briefly, assays were done in 96 well plates where samples were added to buffer with or without the cathepsin inhibitors, CA-074 for cathepsin B and the cathepsin L inhibitor provided with the kit (Z-Phe-Phe-fluoromethylketone). The kit’s assay components are optimized to give maximal activity for their respective enzyme with the specific substrates, but to reduce cross-reactivity of cathepsin B in our samples with the cathepsin L substrate, CA-074 was added to the cathepsin L activity assays in addition to the cathepsin L inhibitor. Following a 10 min incubation at room temperature, the fluorogenic substrates were added to the appropriate experimental wells at a final concentration of 200 µM for both substrates. The plates were then incubated for 2 h at 37 °C. Following incubation, the fluorescence was measured in a GENios Infinite 200pro plate reader and reported as relative fluorescence units per μg of sample (RFU/μg).

### Western Blot

#### Treatment with Concanamycin A

Two T-75 flasks of MB231 cells were grown in DMEM complete for 48 h at which point the media was replaced with DMEM without phenol. 100 nM ConA or DMSO was added to the flasks which were then incubated for 48 h more. The conditioned medium was recovered from each flask, concentrated ~40 times in 10 K cut-off Amicon® Ultra centrifugal filters and frozen for later blotting. Treated cells were harvested via trypsinization, cells were then spun down, resuspended in 200 µl RIPA buffer with protease inhibitors (0.67 nM sodium fluoride, 1 mM sodium orthovanate, 100 nM PMSF), lysed, centrifuged and the supernatant was frozen for later blotting. Protein concentration for all samples was determined by BCA protein assay (Pierce).

#### Western Blotting

10 μg lysate sample or 7.5 μg concentrated conditioned media sample was run on a gel, transferred by Trans-Blot® Turbo™ (Biorad) blotting to a PVDF membrane which was then blocked in 2.5% milk in TBST for 30 min. Blots were next incubated for 1 h with either Cat L (1:2000 dilution) or Cat B (1:1000 dilution) antibodies from R&D Systems. The bottom half of the blots were incubated with antibodies for loading controls; anti-galectin (R&D Systems) (1:2000 dilution) for the conditioned media samples and cyclophilin A (R&D Systems) (1:2000 dilution) for the lysate samples. All antibodies were diluted in 5% BSA in TBST. Following incubation, blots were washed with TBST, incubated with 1:1000 diluted anti-goat secondary antibody (Jackson ImmunoResearch) for 1 h, washed with TBST again and treated with ECL (Millipore). The blots were imaged using the Alpha Innotech Imaging system and the bands were quantified using the Alpha Innotech software.

### Data Analysis

Cathepsin B activity was determined by subtracting RFU (relative fluorescence units) with cathepsin B inhibitor from RFU with no inhibitor. Cathepsin L activity was determined by substracting RFU with cathepsin B and cathepsin L inhibitors from RFU with cathepsin B inhibitor alone. The resulting RFU value was divided by μg loaded for each sample to compare activity across multiple experiments. To quantify the amount of protein seen on the Western blots, the density of bands representing different versions of the cathepsins [pre-pro (55 kDa), pro- (Cat B – 39 kDa; Cat L – 38 kDa) or active (Cat B – 30 kDa; Cat L – 32 kDa) forms] were divided by the density of the loading control bands. JMP 12 software was used to perform an ANOVA followed by the Student t test to statistically analyze the results.

## Results

**Fig. 1:**
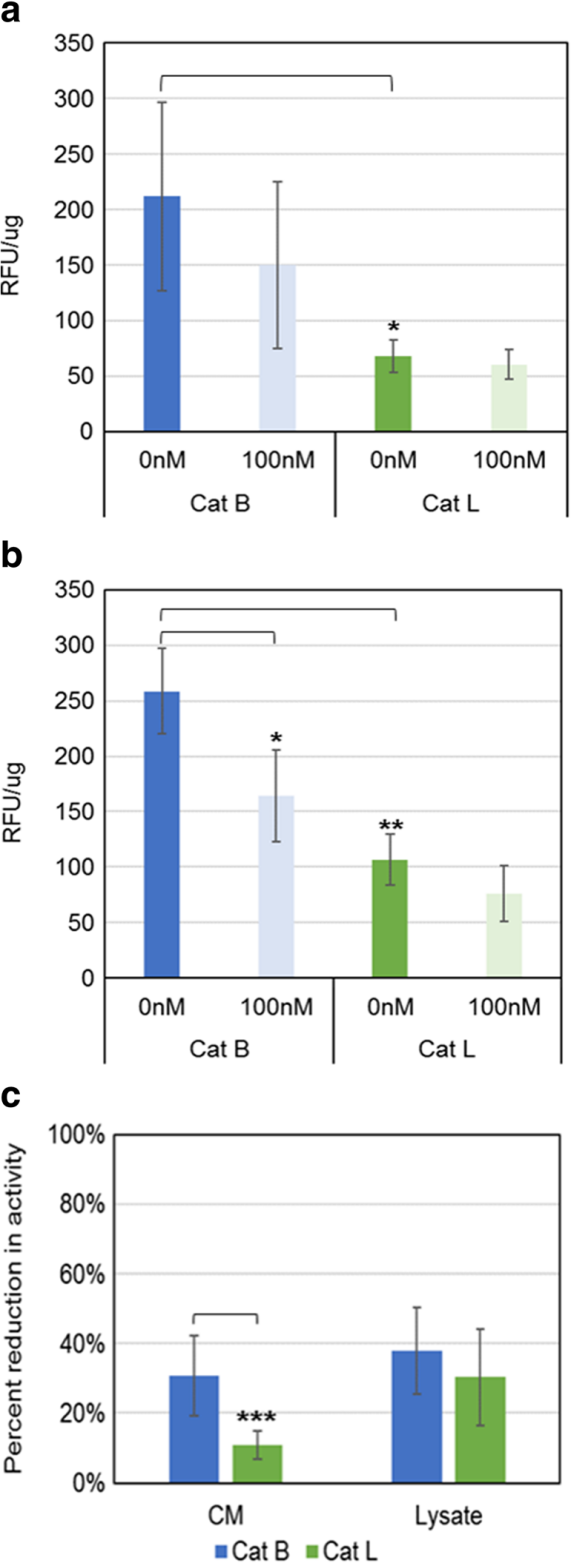
Activity of cathepsins B and L in conditioned media and cell lysates following V-ATPase inhibition. Conditioned media (**a**) and cell lysates (**b**) from cells grown for 24 h in the absence (0 nM) or presence (100 nM) of concanamycin A were assayed for cathepsin activity. *N* = 5, error bars – std. dev, Student t test * *p* < 0.005, ** *p* < 0.0001. (**c**) Percent reduction in cathepsin activity following treatment with concanamycin A in conditioned media (CM) and cell lysates (Lysate). *N* = 5, error bars – std. dev., Student t test *** *p* < 0.05

To determine the activity of cathepsins both within the cell and outside of the cell, MB231 cells were initially grown in complete growth media for 48 h and then the media were replaced with media lacking serum. Concanamycin A (a specific inhibitor of V-ATPases) was added to the cells at this time to a final concentration of 100 nM. Control cells were treated with an equivalent volume of DMSO. Twenty four hours after treatment, the conditioned media were collected and concentrated and the cells were harvested and lysed. The conditioned media and cell lysates were then assayed for cathepsin B and cathepsin L activity using fluorescence-based cathepsin activity assays. As shown in Fig. 1, in the conditioned media the activity of cathepsin B was significantly higher than that of cathepsin L. The activity of cathepsin B was appreciably higher than cathepsin L activity in cell lysates as well. Following treatment with concanamycin A, there was very little change in the activity of cathepsin L in conditioned media, but the activity of cathepsin B trended lower. In addition, treatment with concanamycin A significantly decreased the activity of cathepsin B in cell lysates. The change in cathepsin activity is shown as percent reduction in Fig. 1 **c**. There wasn’t an appreciable difference in the percentage by which concanamycin treatment reduced the activity of cathepsin B versus cathepsin L in cell lysates. However, in the conditioned media, there was significantly greather reduction in cathepsin B activity than reduction of cathepsin L following ConA treatment.

**Fig. 2:**
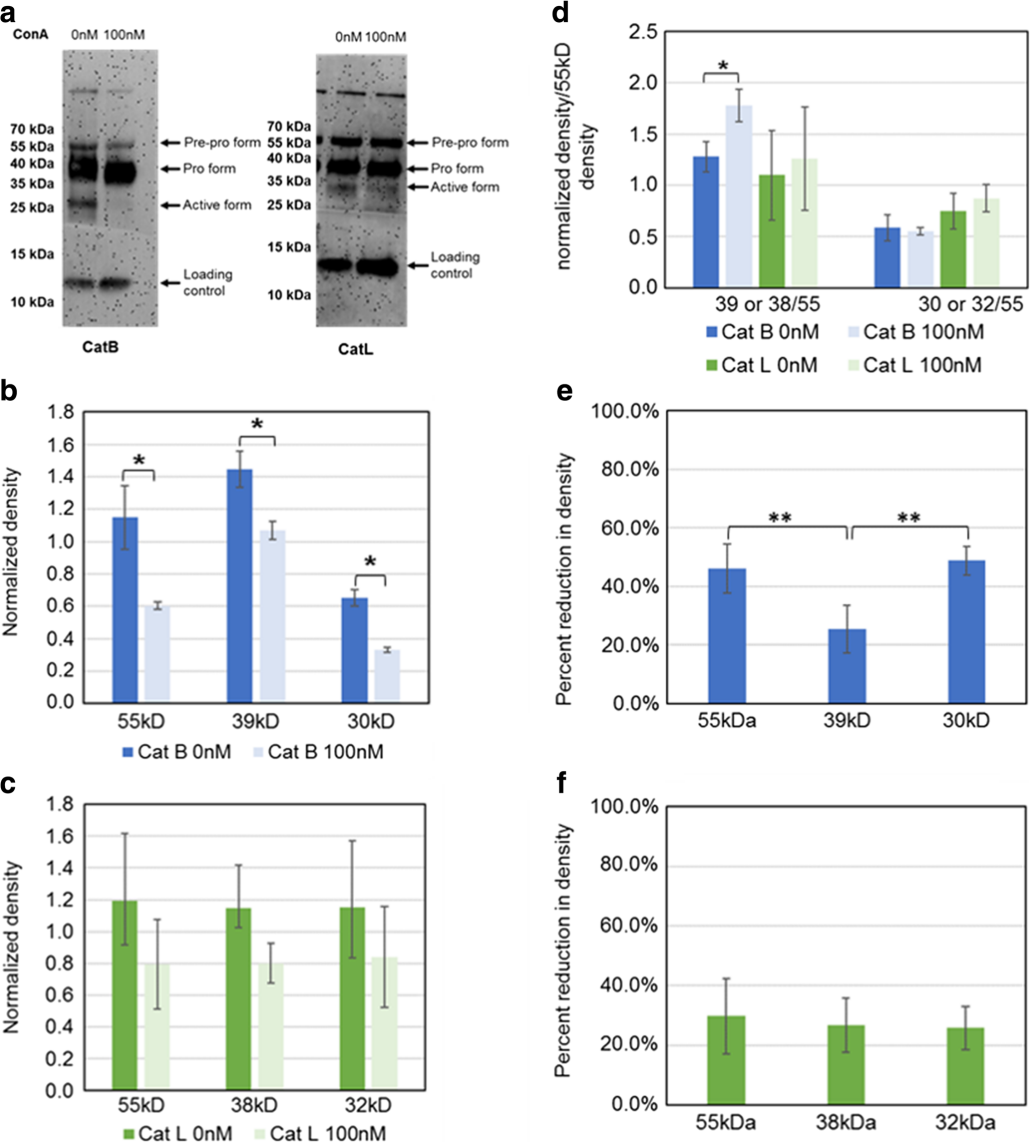
Western blot and quantification of cathepsins B and L in conditioned media following V-ATPase inhibition. Conditioned media from cells grown either in the absence (0 nM) or presence (0 nM) of 100 nM concanamycin A (ConA) were collected, run on a gel and Western blotted with antibodies to cathepsin B (CatB) or cathepsin L (CatL). **a** Representative Western blot, arrows indicate the differing molecular weight forms of each cathepsin or the loading control (galectin). **b** & **c** Quantification of band densities from Western blots for cathepsins B (b) or L (c). The band densities were quantified and normalized to a loading control (galectin). *N* = 4, error bars – std. dev., Student t test * *p* < 0.001. d. Band densities relative to the pre-pro form of cathepsins B or L. The ratio of the pro-form to the pre-pro form (Cat B – 39/55; Cat L - 38/55) or the active form to the pre-pro form (Cat B – 30/55; Cat L - 32/55) was calcualted. *N* = 4, error bars = std. dev., Student t test * *p* = 0.0004. e. & f. Percent reduction of band densities following treatment with concanamycin A for cathepsins B (e) and L (f). *N* = 4, error bars = std. dev., Student t test * *p* < 0.01

Figure 2a shows the Western blot of samples from conditioned media for both Cat B and Cat L. Cathepsins are produced initially in a pre-pro form which is cleaved to a pro-form and directed to the lysosome where they are processed into the final active form. The pro form of Cat B is ~39 kDa in size and the active form is ~30 kDa (indicated by arrows). For Cat L the pro form is ~38 kDa and the active form is ~32 kDa. In these metastatic breast cancer cells cathepsins B and L have been redirected to be secreted from the cell. The pro and active forms of both proteins are present in the conditioned media. In addition, redirecting cathepsins from the lysosome to outside of the cell appears to cause the pre-pro form (~55 kDa for both cathepsins) to be secreted as well.

We quantified the change in the amount of Cat L and Cat B following ConA treatment in both conditioned media and cell lysates. Fig. 2b, c show data for quantification of the Western blots of the conditioned media samples. The band densities were normalized to loading controls. There is a clear decrease in the amount of cathepsin B and cathepsin L following V-ATPase inhibition. For cathepsin B there is a significant decrease in the amount of the 55 kDa, 39 kDa, and 30 kDa forms of the protein. Inhibition of the V-ATPase is known to have general effects on vesicular trafficking within cells [5]. To attempt to differentiate between changes in secretion from changes in the processing/activation of the cathepsins, the density data were divided by the density of the 55 kDa pre-pro form of each cathepsin and then compared in terms of their ratio to the 55 kDa pre-pro form of the proteins (Fig. 2d). We found that for the decrease in cathepsin B activity following V-ATPase inhibition (Fig. 1) there is a corresponding increase in the pro form of Cat B. However, a decrease in the amount of active Cat B was not apparent. For Cat L, there is a slight increase in the active form which does not fit cathepsin L activity seen in Fig. 1. Figure 2e, f show the percent difference in the band densities following V-ATPase inhibition by ConA. The 39 kDa pro form of cathepsin B had significantly less reduction than the pre-pro or active forms of the protein. We believe that reduction in the amount of the pro form of Cat B due to less secretion and processing of the pre-pro form is counter-balanced in part by a decrease in the amount of pro form being converted to the active form of Cat B because of inhibition of cell-surface V-ATPases. This is in line with the increase in the ratio of the pro form to the pre-pro form following ConA treatment seen in Fig. 2d. The secreted forms of cathepsin L all showed about the same percent reduction following V-ATPase inhibition. This likely indicates that the decrease in the amount of these forms in conditioned media is primarily due to inhibition of vesicular trafficking.

**Fig. 3:**
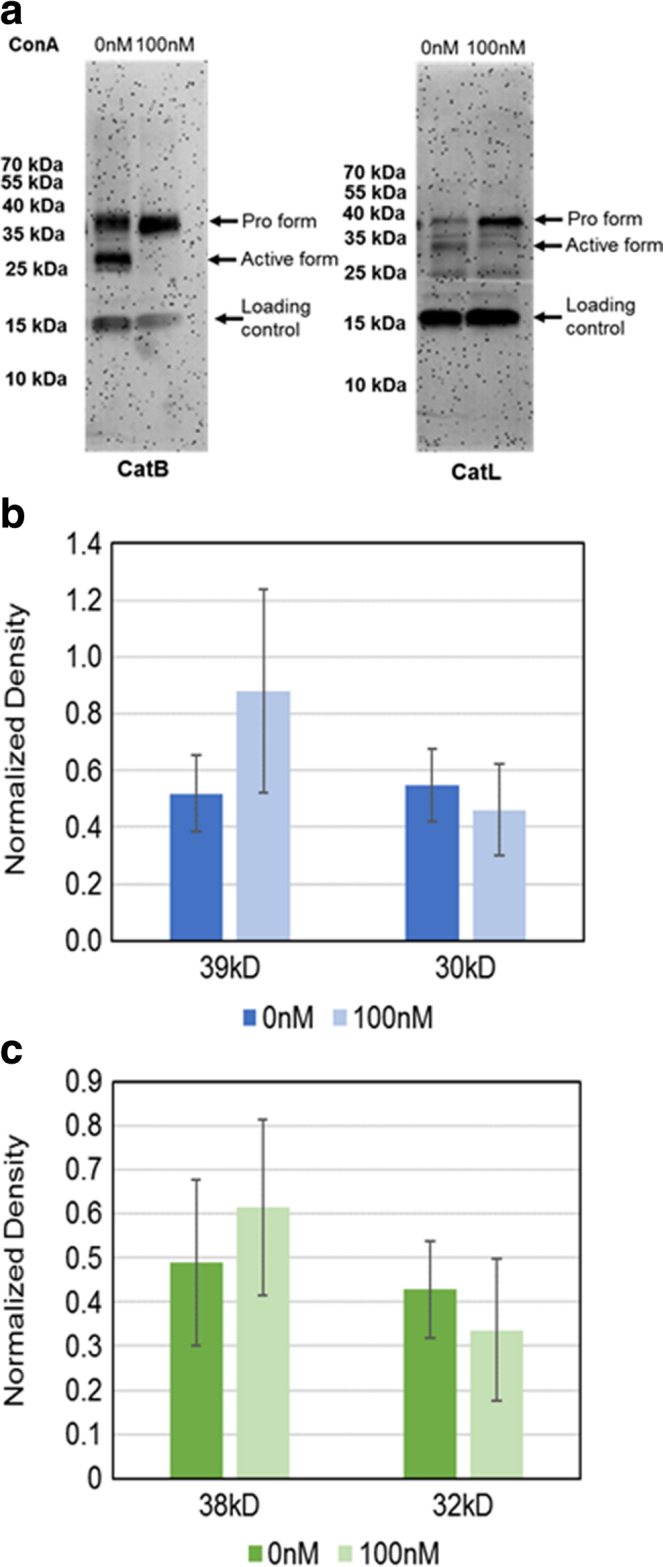
Western blot and quantification of cathepsins B and L in cell lysates following V-ATPase inhibition. Lysates from cells grown either in the absence (0 nM) or presence (0 nM) of 100 nM concanamycin A (ConA) were collected, run on a gel and Western blotted with antibodies to cathepsin B (CatB) or cathepsin L (CatL). **a** Representative Western blot, arrows indicate the differing molecular weight forms of each cathepsin or the loading control (cyclophilin A). **b** & **c** Quantification of band densities from Western blots for cathepsin B (b) or L (c). The band densities were quantified and normalized to a loading control (cylophilin A). *N* = 4, error bars – std. dev

In cell lysates for both Cat L and Cat B there is a decrease in the active form (~32 kDa and ~30 kDa, respectively) of the protein and an increase in the pro-form (~38 kDa and ~39 kDa, respectively) (Fig. 3a). The change in both forms of the cathepsins was quantified and can be seen in Fig. 3b, c.

## Discussion

Several studies have shown that cathepsins are secreted from many cancer cell types [13, 22] and that the presence of elevated levels of secreted cathepsins correlates with a poor prognosis for the spread of cancer [13, 14]. Cathepsins in the extracellular space are thought to facilitate invasion by breaking down parts of the extracellular matrix or by activating other proteases that degrade extracellular matrix proteins [13] and studies that look at the effects of inhibiting cathepsins see a decrease in invasion [23]. However, there are very few papers that explore how typically intracellular cathepsins are targeted for secretion and activated by the cancer cells. This study aimed to shed light on the latter, by determining if the activity of V-ATPases affects the activity of cathepsins secreted from breast cancer cells.

Cathepsins are intracellular proteases typically found in endosomes and lysosomes. They are trafficked from the Golgi in a pro form that does not have enzymatic activity. Cathepsins are processed into the active form once they reach a compartment with an acidic lumen via autoproteolysis which is triggered by low pH [11]. Since cathepsins need an acidic environment to be active it is intriguing to think about how secreted cathepsins are activated. One could assume that they are activated in secretory vesicles, however there are problems with this assumption. Cathepsins are non-specific proteases and thus they are kept in an inactive pro form until they reach the lysosome to prevent them from degrading other proteins as they travel through the endomembrane system. Thus if they were activated in secretory vesicles they would likely degrade other proteins destined for secretion. The other possibility is that they are activated after they have reached the extracellular space. This is perhaps the best way to ensure that there isn’t off-target degradation of secreted proteins. V-ATPases are vitally important for acidifying intracellular compartments and they are necessary for activating lysosomal cathepsins. Previous research has shown that V-ATPases are present on the cell membrane of metastatic breast cancer cells [9, 10] and inhibiting their activity decreases the invasiveness of those cells. Therefore, we hypothesize that cathepsins are activated soon after reaching the cell surface in a localized low pH environment created by V-ATPases at the cell surface.

We decided to explore the possibility of a link between V-ATPases at the cell membrane of metastatic breast cancer cells and the activation of secreted cathepsins. Our data show that unprocessed forms of cathepsins B and L are being secreted from MB231 cells (Fig. 2), thus there is a significant amount of activation that needs to occur in the extracellular space. We hypothesized that inhibiting V-ATPases would cause a decrease in the amount of the active form of cathepsins B and L along with a decrease in the activity of both cathepsins in the extracellular space. We found that overall there was more cathepsin B in the MB231 cells than cathepsin L and the activity of cathepsin B was considerably higher than cathepsin L in the extracellular space. When V-ATPases were inhibited in MB231 cells, the activity of secreted cathepsin B decreased and there was a corresponding increase in the pro form of cathepsin B in conditioned media (Figs. 1a and 2). This data supports our hypothesis that V-ATPases are involved in the activation of extracellular cathepsins. This is in line with the widely accepted notion that secreted cathepsin B is playing a role in cancer metastasis [2, 16, 21]. Due to its cancer promoting effects, cathepsin B has been proposed as a target for cancer therapeutics [2, 12, 23]. V-ATPases have also been linked to cancer metastasis. Our findings provide a link between these two proteins that could be exploited for the development of effective and selective treatments to impede cancer metastasis. Surprisingly, there didn’t seem to be much effect on activation of secreted cathepsin L.

The role that each of these cathepsins is playing in invasion is an open question. Recently Kubisch et al. [24] showed that V-ATPase inhibition lead to relocalization of pro-cathepsin B to the extracellular space in MCF7 cells. However, they did not see mature active cathepsin B in the conditioned media from these poorly metastatic cells whereas we see abundant mature cathepsin B in conditioned media from the highly metastatic MB231 cells (Fig. 2). Interestingly, MB231 cells have both intracellular and cell surface V-ATPases whereas MCF7 cells only have intracellular V-ATPases [9]. Previous data have shown that inhibiting V-ATPases inhibits invasion [9, 10] and our experiments indicate that inhibiting V-ATPases decreases the activity of secreted cathepsin B. Activating cathepsin B in the extracellular space may be part of the mechanism by which cell-surface V-ATPases are enhancing invasion of breast cancer cells. The activity of cathepsin L in the extracellular space may promote cell invasion through a mechanism that does not involve V-ATPases.

Our future experiments will continue to clarify the role of individual cathepsins in V-ATPase-mediated invasion, by exploring if there is specificity in which pool of V-ATPases is activating cathepsin B. The a subunit of V-ATPases comes in four isoforms in humans (a1-a4) and two of the isoforms, a3 and a4, have been shown to localize at the plasma membrane of MB231 cells [10, 25]. These isoforms also play a role in V-ATPase-mediated invasion [10]. We plan to determine which of these isoforms is involved in the activation of cathepsin B. Additionally, we will explore the interplay of cathepsin L and cathepsin B activity in the extracellular space and their relative importance for breast cancer cell invasion.

## Conclusion

This study aimed to help fill in the gaps in our understanding of the molecular mechanisms that underly the role of secreted cathepsin B and cathepsin L in cancer metastasis and to determine if there is a link between plasma membrane V-ATPases and the activity of these secreted cathepsins. The role that V-ATPases at the cell surface are playing in cancer metastasis is not clearly understood. A fuller understanding of these molecular mechanisms is important for the successful development of cancer therapies that target V-ATPases as well as cathepsins B and L without undo side-effects or poor efficacy [2]. This study taken with the results from Kubisch et al. [24] provides support for a link between cell-surface V-ATPases and activation of extracellular cathepsins, an important next step in elucidating the role of cell surface V-ATPases in cancer metastasis.
